# Transcriptome profiling reveals novel insights into the regulation of calcium ion and detoxification genes driving chlorantraniliprole resistance in *Spodoptera exigua*

**DOI:** 10.1016/j.heliyon.2024.e40556

**Published:** 2024-11-22

**Authors:** Changhee Han, Juil Kim

**Affiliations:** a Interdisciplinary Graduate Program in Smart Agriculture, Kangwon National University, Chuncheon, Republic of Korea; b Agriculture and Life Sciences Research Institute, Kangwon National University, Chuncheon, Republic of Korea

**Keywords:** *Spodoptera exigua*, Chlorantraniliprole, Calcium ion imbalance, Endoplasmic reticulum stress, RNA sequencing

## Abstract

Since the commercialization of diamide insecticides, including chlorantraniliprole, in 2007, the overuse of diamide insecticides for over a decade has resulted in excessive chlorantraniliprole resistance in *Spodoptera exigua*, causing continuous economic losses. While RyR target-site mutations and detoxification enzymes such as cytochrome P450 have been studied as the leading causes of resistance, previous studies, including functional research and synergistic tests, have not confirmed a clear correlation between these factors and the development of resistance. Thus, transcriptome analysis was employed to investigate alternative strategies beyond mutation(s) in RyR or metabolic factors involving detoxification pathways that allow diamide-resistance *S. exigua* to counteract the calcium ion imbalances induced by chlorantraniliprole effectively. Diamide-resistant, susceptible strains and its F1-hybrid of *S. exigua* were used for the RNAseq-based differentially expressed gene (DEG) analysis. In total 4669 genes were differentially expressed, with 2809 upregulated and 1860 downregulated in the resistant strain compared to the susceptible strain. GO, KEGG enrichment and orthologous analyses demonstrated that genes involved in metabolic factors were overrepresented in the resistant strain. In particular, overexpressed endoplasmic reticulum (ER)-related calcium ion homeostasis and cell stability-associated genes were newly identified in resistant strain. The selected differentially expressed genes were validated then with qPCR. These genes were inferred to induce cell stability to overcome ER stress derived from calcium ion imbalance caused by chlorantraniliprole. These results provide advanced insights into the critical roles of calcium ion homeostasis- and cell stability-related genes in conferring diamide insecticide resistance.

## 1 Introduction

The beet armyworm, *Spodoptera exigua* (Hübner) (Lepidoptera: Noctuidae), is a polyphagous insect pest that causes significant damage to crop yields [1,2]. It originated in East Asia and now inflicts severe economic damage worldwide [[3], [4], [5]]. Since the introduction of flubendiamide, a diamide insecticide, in 2007, other diamide insecticides, such as chlorantraniliprole, have been developed and commercialized as anthranilic diamide [6,7]. These insecticides are highly specific and effective against lepidopteran pests and have significantly lower mammalian toxicity, making them some of the most widely used insecticides to date [8,9].

Diamides act by specifically binding to ryanodine receptors (RyR), which are calcium ion channels predominantly found in the endoplasmic reticulum (ER) and sarcoplasmic reticulum of insect muscle cells [10,11]. This binding induces excessive calcium ion imbalance, leading to insect pest mortality [12,13]. However, over the past decade, 2010, the overuse of chlorantraniliprole has led to the development of insecticide resistance in *S. exigua*, which was reported in China [[14], [15], [16]]. In 2019, resistant ratio from bioassay results of chlorantraniliprole to field *S. exigua* showed up to 2477-fold [17]. Similarly, in Korea, resistance to diamide insecticides has been reported since 2017, and *S. exigua* in Korea has rapidly developed resistance against the diamide insecticides. Bioassay results showed a decrease in effectiveness by over 200,000-fold, significantly reducing mortality among the field populations, thus becoming devastating lepidopteran pest [18,19]. Several studies have reported that amino acid substitutions at target sites of chlorantraniliprole, RyR, such as I4790M and G4946E contribute to diamide insecticide resistance by reducing the binding affinity with chlorantraniliprole [[20], [21], [22], [23], [24], [25]]. Among the reported mutations, G4946E has been functionally proven to be highly related to the development of resistance, while I4790M mutation contributes minimally to the development of diamide resistance [[26], [27], [28]]. Furthermore, a recent study has reported that only the I4790M mutation in RyR was identified in diamide-resistant *S. exigua* populations in Korea and that this mutation had an unstable correlation with resistance [18,19]. Additionally, many studies have focused on detoxification enzymes involved in the phases of xenobiotics metabolism to elucidate the development of diamide resistance [[29], [30], [31]]. Results from synergism test have presented that cytochrome P450s belonging to phase I (functionalization) plays a significant role in developing resistance [[32], [33], [34]]. However, this information remains insufficient to elucidate the excessively developed resistance.

Based on the results of previous studies, we hypothesized that changes in the expression of genes involved in detoxification–specifically Phase I enzymes such as cytochrome P450 (CYP) and carboxyl/cholinesterase (CCE), Phase II (conjugation) enzymes like glutathione S-transferase (GST) and UDP-glycosyltransferase (UGT), and Phase III (excretion) enzyme ATP-binding cassette transporter (ABC)–plays a critical role in the decomposition of diamides. Moreover, we further hypothesized that there were possibly additional metabolic activities beyond these detoxification phases that counter the cellular calcium ion imbalance induced by chlorantraniliprole.

Despite the economic importance of *S. exigua*, studies on the metabolic genes’ expression differences between diamide-resistant and susceptible strains over generations, and the identification of resistance-inducing candidate genes, remain unclear. Therefore, RNA deep sequencing of *S. exigua* was performed in this study to obtain a *de novo* transcriptome, generate unigenes, expression profiles, and identify diamide resistance-specific genes. This included analyzing known xenobiotic detoxification enzymes as well as exploring genes involved in overcoming the calcium ion imbalance caused by chlorantraniliprole.

To compare differential gene expression between resistant and susceptible strains, we also included their F1-hybrid in the assembly and clustering process to generate unigenes, providing a robust framework for interpretation. We classified genes through orthologous classification, functional enrichment analysis, and differential expression gene (DEG) analysis. Further, we compared the relative expression levels of the classified diamide resistance candidate genes with the resistance ratio observed in the *S. exigua* field population. Additionally, we inferred the detailed mechanisms contributing to resistance, including protein–protein interactions.

## 2 Materials and methods

### 2.1 Insects

An insecticide-susceptible strain of *S. exigua*, beet armyworm, BAW (BAW-S) was maintained over four years in the Insect molecular toxicology lab at Kangwon National University [35]. A diamide-resistant strain (BAW-R) was selected and maintained from among individuals that survived treatment with chlorantraniliprole (DuPont™ Altacor® 5 %, WG, water dispersible granule [35]. Insects were reared under controlled conditions at a temperature of 25 ± 1 °C and a relative humidity of 60 ± 5 %, and a photoperiod regime of 14 h of light and 10 h of darkness. Larvae were fed an artificial diet and the basic rearing conditions were the same as previously reported [19]. F1 hybrids were produced by single-pair mating of resistant and susceptible adults (15 pairs of R♂ × S♀, BAW-RS) as previously reported [35]. Field populations of *S. exigua* larvae were collected from three different localities in the Korean Peninsula, where cabbage and green onion crops were cultivated. The Jindo population was collected in June 2021 (JD, 34°25′13.3″N 126°10′23.4″E), the Nonsan population was collected in June 2023 (NS, 36°09′03″N 127°09′48″E), and the Chuncheon population was collected in July 2023 (CC, 37°56′19″N 127°47′02″E) as previously reported [19,34].

### 2.2 Preparation of samples and sequencing

For RNA-seq sample preparation, the beginning of the 5th-instar was set as close as possible to the bioassay survey period to ensure consistency in larval development. RNA was extracted from BAW-S, BAW-RS and BAW-R strains for RNA-seq and subsequent cDNA synthesis, with each strain's development period consistently determined. Field population samples of JD, NS and CC from 5th-instar larvae within 12 h of molting after one generation, were selected for RNA extraction for subsequent cDNA synthesis. Each sample contained five larvae as a biological replicate. RNA was extracted using the RNeasy Mini Kit (Qiagen, Hilden, Germany), according to the manufacturer's instructions. RNA was validated and quantified using an Agilent 2200 TapeStation (Agilent Technologies, Santa Clara, CA, USA), and RNA integrity was confirmed by electrophoresis on a 1 % agarose gel. RNA-seq libraries were prepared using the TruSeq RNA Sample Prep Kit v2 (Illumina, San Diego, CA, USA). Samples were sequenced on the Hiseq4000 platform using TruSeq 3000/4000 SBS Kit v3 (Macrogen, Seoul, Korea).

### 2.3 Raw reads and preprocessing

Paired-end raw reads of nine samples in FASTQ format were obtained from Illumina sequencing. The raw reads were processed using Trimmomatic v0.39 [36] to remove low-quality sequences and adapter sequences using the parameters LEADING:3, TRAILING:3, SLIDINGWINDOW:4:20, MINLEN:36. To further eliminate potential contaminants, Kraken2 v2.1.2 was employed to remove sequences identified as bacterial, viral, human, or fungal RNA [37]. The quality of the resulting clean reads was assessed using FastQC v0.11.9 (http://www.bioinformatics.babraham.ac.uk/projects/fastqc), confirming the high quality of the processed reads.

### 2.4 Assembly and construction of unigenes

The clean reads were assembled *de novo* using the short-read assembler Trinity v2.13.2 [38]. The assembled sequences were then clustered and simplified using CD-HIT-EST from the CD-HIT v4.8.1 package, with a similarity threshold of 90 % to reduce dataset complexity and improve analysis efficiency [39]. Unigenes for each strain (susceptible, diamide-resistant and F1-hybrid) were then generated. These unigenes were further clustered to create a reference unigene set for the reference genome. Additionally, all clustered unigenes were processed with TransDecoder (v5.7.1; https://github.com/TransDecoder/TransDecoder) to predict the longest open reading frames (ORFs) and obtain the corresponding protein sequences.

### 2.5 Functional annotation

Unigenes were aligned and annotated using OmicsBox (v3.1.11; https://www.biobam.com/omicsbox), InterProScan v5.67–99.0 and DIAMOND v2.1.8 to classify contigs according to the NCBI Insecta non-redundant (nr) protein database, protein domains, and the three Gene Ontology (GO) categories: biological process, cellular component, and molecular function (E-value: 1e-5) [40]. For the Kyoto Encyclopedia of Genes and Genomes (KEGG) pathway annotation, we utilized the KAAS v2.1 online software, employing the SBH method and Eukaryotes + bmor, bman, haw, tnl, and pxy gene set parameters [41].

### 2.6 Profiling differential genes expression

The resistant and susceptible reads were mapped to the reference unigene using Bowtie2 v2.4.4 and RSEM v1.3.1 to estimate the count abundance [42]. The resulting read counts matrix was normalized using the TMM method to effectively adjust for RNA composition differences across samples and minimize the influence of outliers by excluding extreme values. Contigs were excluded if their count value was less than 2 in at least two out of three replicates for each strain. Pairwise differential expression analysis between resistant and susceptible strains was conducted using edgeR v4.0.16 [43]. The Benjamini-Hochberg procedure was applied to control false positives due to multiple comparisons [44]. Genes were judged as differentially expressed if they had FDR <0.01 and |Log2FoldChange| > 2. Additionally, genes were required to have a log2CPM value > 2 to ensure sufficiently high expression levels for comparison. The enrichment analysis for DEGs in terms of GO and KEGG pathways was performed using Fisher's exact test (p < 0.05).

### 2.7 Analysis of co-expression interaction networks

To analyze gene-gene interactions, differentially expressed candidate genes potentially related to diamide insecticide resistance were examined using the STRING v12.0 online software to predict co-expressed functional partner proteins (database: Lepidopteran, FDR <0.05, default parameters) [45]. Owing to the limited availability of the *Spodoptera exigua* database in STRING, the *Heliothis virescens* database, which has the highest identity percentage, was used as a substitute.

### 2.8 Analysis of orthologous clusters

Orthologous and specific genes among the predicted protein unigenes of the three different strains were examined and visualized using Orthovenn3 online software [46]. The comparative analysis and visualization of orthologous gene clusters and unique genes specific to each strain were conducted using Venn diagrams.

### 2.9 Quantitative real-time PCR

To validate the expression of differentially expressed candidate genes, complementary DNA (cDNA) was synthesized from the total RNA extracted from susceptible, resistant, and field populations using a RNeasy Plus Mini Kit (Qiagen) and Superscript III cDNA Synthesis Kit (Enzynomics, Daejeon, Korea). Quantitative real-time PCR (qPCR) was performed using specific primers for each target candidate gene and glyceraldehyde 3-phosphate dehydrogenase (GAPDH) as the reference gene (Table S13). qPCR was conducted using the Topreal™ SYBR Green qPCR PreMIX (Enzynomics) on the ABI QuantStudio 1 (Thermo Fisher Scientific Inc., Waltham, MA, USA) platform.

qPCR was performed in triplicate in a total volume of 20 μL, following the protocol provided by the manufacturer. Each reaction contained 1 μL of template cDNA at a concentration of 50 ng/μL, 1 μL each of forward and reverse primers diluted to 10 pmol/μL, 10 μL of Topreal™ SYBR Green qPCR PreMIX, and 7 μL of ddH_2_O. The amplification efficiency was assessed by melting curve analysis. The relative expression levels were normalized to GAPDH expression in the same samples and calculated using the 2^−ΔΔCT^ method.

### 2.10 Statistical analysis

SAS software (SAS Institute 9.4, Cary, NC, USA) and R language were used for statistical analyses. To identify significant differences between strains, Shapiro-Wilk test and Leven's test were conducted to evaluate the normality and homogeneity of variances, respectively (p < 0.05 was considered statistically significant). Then, we employed One-way ANOVA followed by Tukey's HSD test. Significant difference was represented by asterisk (∗p < 0.05, ∗∗p < 0.01, ∗∗∗p < 0.001). The p-values for the functional enrichment analysis were determined using Fisher's exact test. Statistical details are described in Figure legends, and the graphs were generated using SigmaPlot software and R language.

## 3 Results

### 3.1 Sequences statistics

Illumina sequencing generated raw reads for three distinct samples: susceptible (BAW-S), resistant (BAW-R), and F1-hybrid (BAW-RS), each with three biological replicates. The total number of raw reads per sample ranged from 117 million to 157 million (Table S1). The total raw read bases varied between 11 Gb and 16 Gb (Table S1).

Following the removal of adapters and low-quality reads, the percentage of retained reads was >95 % for all samples, indicating high sequencing quality (Table S1). Contaminant RNA was subsequently removed to produce clean reads. The final read bases ranged from 10 Gb to 12 Gb, resulting in reduced variability in read bases among the samples (Table S1). This consistency in read bases ensures the reliability of the sequencing data for subsequent analysis.

### 3.2 De novo assembly of high-quality unigene sets

To reduce errors and ensure an accurate depiction of gene expression patterns, we performed *de novo* assembly analysis after preprocessing, using samples from susceptible strain, resistant strain, and F1-hybrid to construct the transcriptomes. Nine clean reads were assembled, generating 67,865–94,149 contigs with a total length of 70–119 Mb (Table S2). The average contig length ranged from 867 to 1272 bp, and N50 length varied from 1363 to 2379 bp (Table S2). The GC content of the contigs was between 39.51 and 41.83 % (Table S2).

**Fig. 1:**
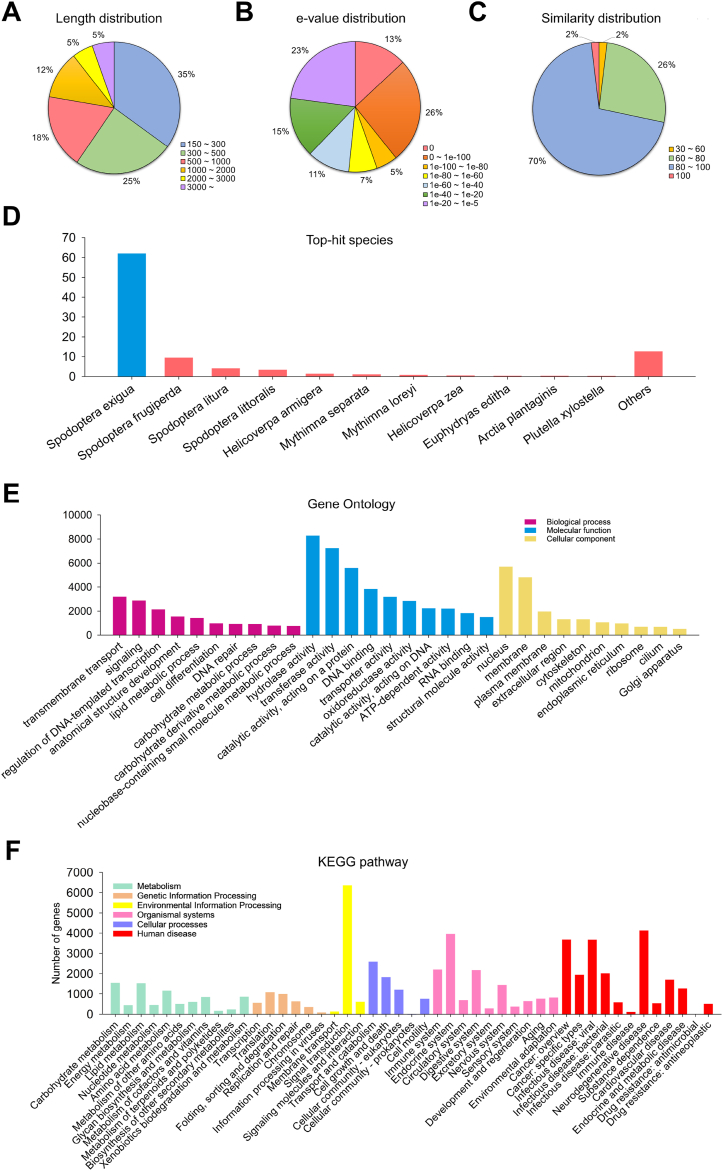
Annotation statistics and results from BLAST, GO, and KEGG analyses establishing the reliability and gene distribution of the reference unigene set. (A) Pie chart presenting the length distribution of the reference unigene created after clustering. (B) Pie chart presenting the distribution of e-values derived from the BLASTX results of the reference unigene. (C) Pie chart presenting the percent identity of BLASTX results of the reference unigene. (D) Bar plot showing the distribution of top-hit species as shown by BLASTX results of the reference unigene. (E) GO-slim distribution classified into three main categories of reference unigene: biological process, cellular component, and molecular function. (F) Second hierarchy distribution of KEGG pathway classified into six categories: metabolism, genetic information, processing, environmental information processing, cellular processes, organismal systems, and human disease.

The assembly results of all samples were clustered by their respective strain, generating strain-specific unigene. Additionally, clustering of all assemblies produced a high-quality reference unigene set. The length distribution of the reference unigene showed the highest proportions in the ranges of 150–300 bp (35 %, 63,129 contigs), 300–500 bp (25 %, 44,232 contigs), and 500–1000 bp (18 %, 32,753 contigs) (Fig. 1A, and Table S3). The BUSCO assessment of the reference unigene set, used as the reference genome, demonstrated high quality with a completeness score of 98.1 %, indicating its suitability for further analyses (Table S4). The longest open reading frames (ORFs) were identified for each unigene to predict the protein-coding sequences. These protein-coding unigenes were then generated for subsequent analyses. Detailed statistics are presented in Table S5.

### 3.3 Functional annotation of reference unigenes

Before investigating the gene expression differences between resistant and susceptible strains, we functionally annotated the reference unigenes using Gene Ontology (GO) and Kyoto Encyclopedia of Genes and Genomes (KEGG) pathway.

A total of 79,623 contigs (44 %) were successfully aligned with the NCBI Insecta non-redundant (nr) protein database (Table S6). The distributions of e-value, similarity percentage, and the significant top species hits in the BLAST analysis are presented in Fig. 1A–D, and Table S6. Subsequently, the reference unigenes were categorized in three GO categories: biological process, cellular component, and molecular function, with 53,715 contigs (30 %) successfully annotated (Table S6). Fig. 1E shows the top 10 subcategories of each GO category. Notable GO terms included “transmembrane transport” (6 %, 3225), “signaling” (5 %, 2912), and “regulation of DNA-templated transcription” (4 %, 2182) in biological processes; “hydrolase activity” (15 %, 8294), “transferase activity” (14 %, 7265), and “catalytic activity, acting on a protein” (10 %, 5600) in molecular functions; and “nucleus” (11 %, 5708), “membrane” (9 %, 4834), and “plasma membrane” (4 %, 2010) in cellular components.

Furthermore, KEGG pathway annotation of the reference unigenes categorized the genes into six pathway categories: metabolism, genetic information processing, environmental information processing, cellular processes, organismal systems, and human diseases. In total, 60,234 genes were classified into the second hierarchy of the KEGG pathway (Table S7). Notable pathways included “carbohydrate metabolism” (3 %, 1576) under metabolism, “Translation” (2 %, 1123) under genetic information processing, “Signal transduction” (11 %, 6362) under environmental information processing, “Transport and catabolism” (4 %, 2610) under cellular processes, “Endocrine system” (7 %, 3972) under organismal systems, and “Neurodegenerative disease” (7 %, 4143) under human diseases (Fig. 1F, and Table S7).

This preliminary analysis offered an extensive overview of the biological functions and pathways related to our reference unigene, establishing as a foundation for subsequent analyses.

### 3.4 Differentially expressed genes (DEGs) and functional analysis

**Fig. 2:**
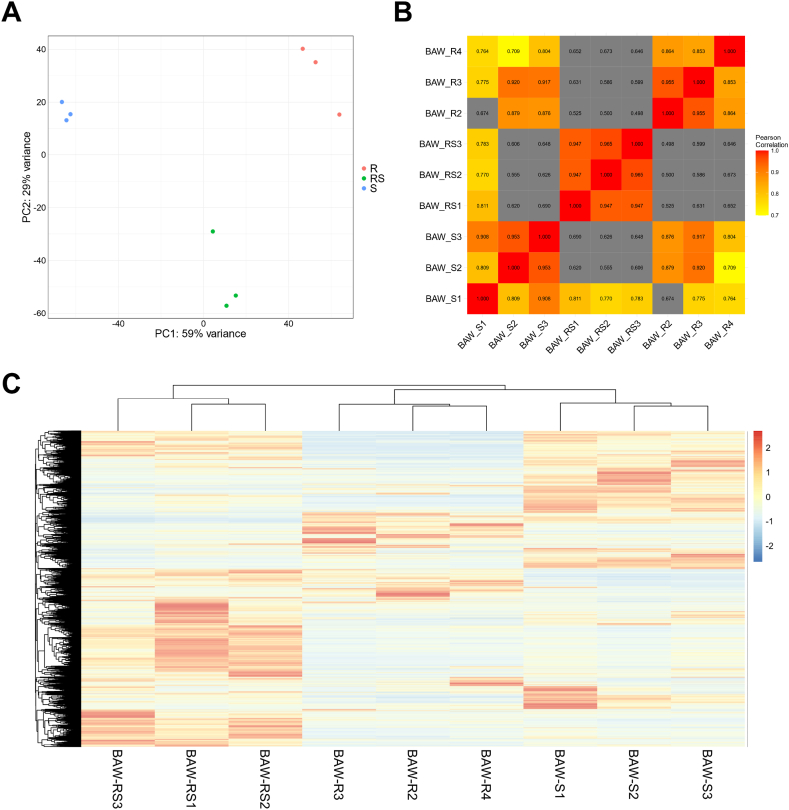
Comparative expression profile analyses revealing gene expression patterns across samples from the three strains. (A) Principal component analysis plot illustrates the variance in gene expression profiles among the F1-hybrid (RS), resistant (R) and susceptible (S) strains. Different colors represent different groups: red, R; green, RS; and blue, susceptible strains. (B) The heatmap represents the Pearson correlation coefficients among the samples. Higher correlation values indicate more similar gene expression profiles. The color gradient from red to yellow (1.0–0.7) represents the higher and lower correlations; low correlations are indicated by gray. (C) The heatmap presents the hierarchical clustering of gene expression profiles across all samples. Each gene expression value was normalized to the Z-score.

To examine the DEGs between susceptible and resistant strains, the reads from each pre-processed sample were aligned to reference unigene. This process generated a raw counts matrix which can be found in Table S8. The results showed that at least 92 % of the clean reads aligned at least once in all samples, indicating a high alignment rate (Table S9). A principal component analysis (PCA) plot generated based on read counts from aligning each strain's sample reads to the reference unigenes. The plot clearly showed that each strain possessed its own unique spaces, indicating the presence of genetic and expression variability underlying their resistance or susceptibility to the diamide insecticide (Fig. 2A). Pearson correlation analysis was performed to quantify the relationships between samples, revealing various gene expression profiles corresponding to different levels of susceptibility to the diamide insecticide (Fig. 2B). In addition, a heatmap was generated using the read counts of all samples, representing the comprehensive gene expression patterns (Fig. 2C).

**Fig. 3:**
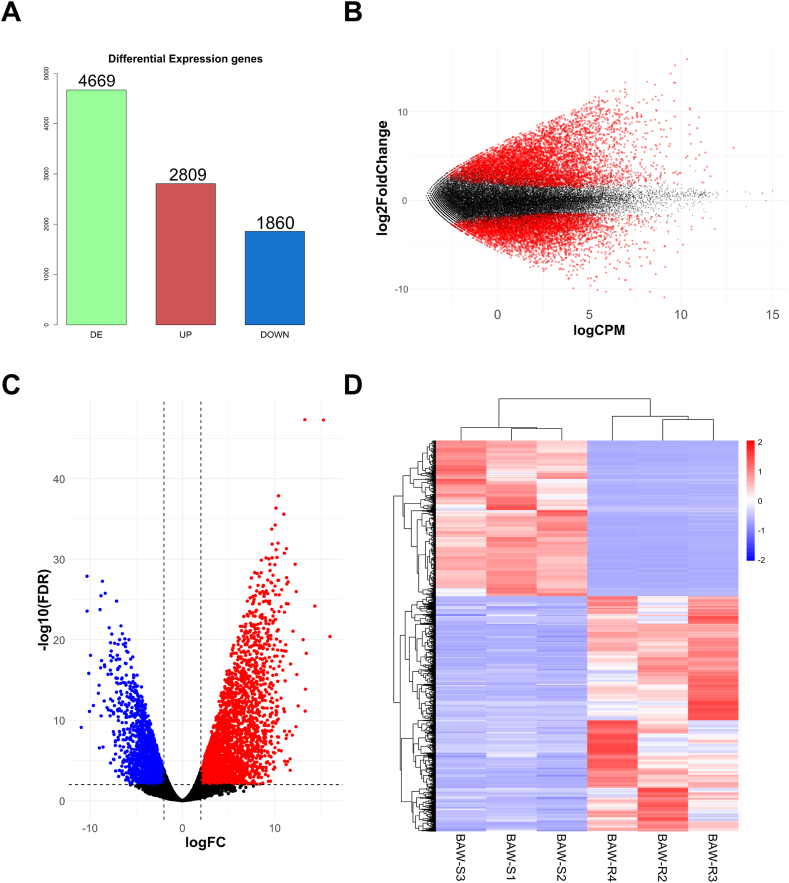
Analysis of differentially expressed genes between beet armyworm susceptible (BAW-S) and resistant (BAW-R) strains. (A) Bar plot presents the number of differentially expressed (DE) genes, with a total of 4669 genes identified. Among these, 2809 genes are upregulated (UP), and 1860 genes are downregulated (DOWN), shown in red and blue, respectively. (B) MA plot visualizes the log2foldchange (logFC) against the average expression levels (calculated as logCPM) of genes. Red dots indicate significant differentially expressed genes (| logFC | > 2, FDR <0.01), Black represents non-significance. (C) Volcano plot represents differential gene expression of the resistant strain against the susceptible strain. Red and blue dots indicate upregulated and downregulated genes, respectively (| logFC | > 2, FDR <0.01). Black indicates non-significant. (D) Heatmap shows the differential gene expression profiles across a total of six resistant and susceptible sample (| logFC | > 2, FDR <0.01). Gene expression values normalized by the Z-score are expressed in colors from red to blue.

Read counts normalized using the trimmed mean of M values (TMM) method were compared between resistant and susceptible strains; in total, 4669 genes were found to be differentially expressed, including 2809 upregulated and 1860 downregulated genes with |log2foldchange| > 2, false discovery rate (FDR) < 0.01, and log2CPM >2 criteria. (Fig. 3A–D, and Table S10). To identify the occupied functions of DEGs, we conducted GO and KEGG pathway enrichment analyses using Fisher's exact test (FDR <0.05). In the GO term enrichment analysis, significant enrichment was observed for “catalytic activity, acting on a protein,” oxidoreductase activity, transferase activity, and structural molecule activity (Fig. 5A). In the KEGG pathway enrichment analysis, the top pathways included “Drug metabolism– cytochrome P450”, and “Metabolism of xenobiotics by cytochrome P450” belonging to xenobiotics biodegradation and metabolism. Additionally, pathways such as “Fatty acid degradation” belonging to lipid metabolism and “Glutathione metabolism” under metabolism of other amino acids were highly enriched (Fig. 5B).

These results underline significant differences in the expression levels of genes involved in pathways related to the detoxification and xenobiotic metabolism, which could be associated with diamide resistance in *S. exigua.*

### 3.5 Clustering of strain-specifically expressed genes

**Fig. 4:**
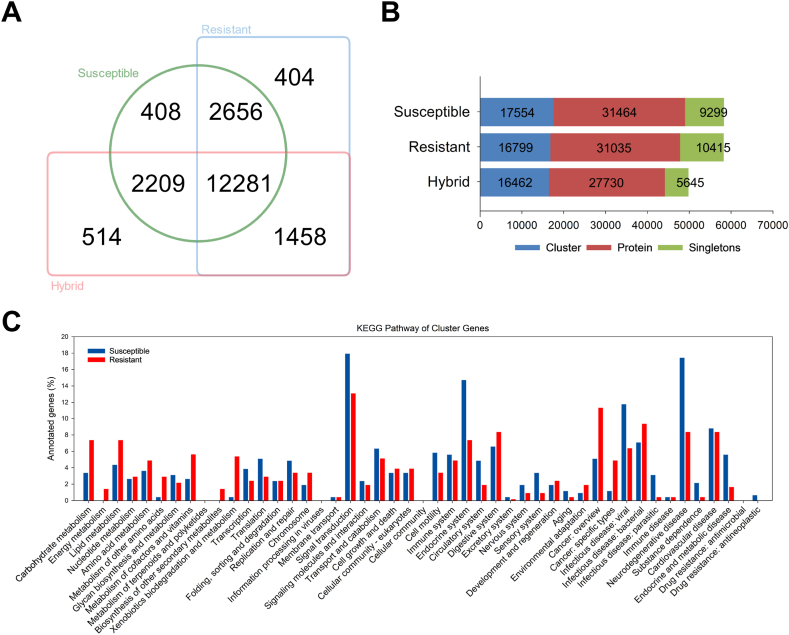
Analysis of orthologous protein cluster and Kyoto Encyclopedia of Genes and Genomes (KEGG) pathway analysis. (A) Edwards-Venn diagram represents the distribution of orthologous protein clusters among hybrid, resistant and susceptible strains. The overlap areas indicate shared orthologous proteins, while the other areas indicate unique orthologous proteins. (B) Summary of the number of clusters, proteins, and singletons across three strains. shown in a bar plot. (C) Bar plot depicting the ratio and distribution of unique proteins between the resistant (red) and susceptible (blue) strains, annotated in the second hierarchy of the KEGG pathway.

We used Orthovenn3 to evaluate protein sequences of each unigene for unique proteins linked to diamide resistance. Comparative analysis revealed a total of 19,930 clusters, consisting common and unique gene sets (Fig. 4A). This analysis produced 17,554 clusters for the susceptible strain, 16,799 clusters for the resistant strain, and 16,462 clusters for the F1-hybrid (Fig. 4B). Among the three strains, 12,281 clusters were found as common cluster. Additionally, 14,937 clusters were shared between the susceptible and resistant strains, 14,490 clusters were shared between the susceptible strain and F1-hybrid, and 13,739 clusters were shared between the resistant strain and F1-hybrid (Fig. 4A). The number of unique clusters identified was 408 in the susceptible strain, 404 in the resistant strain, and 514 in the F1-hybrid (Fig. 4A). The genes within these unique clusters were further analyzed for examining their biological pathways using KEGG pathway analyses (Table S7). Fig. 4C represents an illustration based on the results from those analysis.

**Fig. 5:**
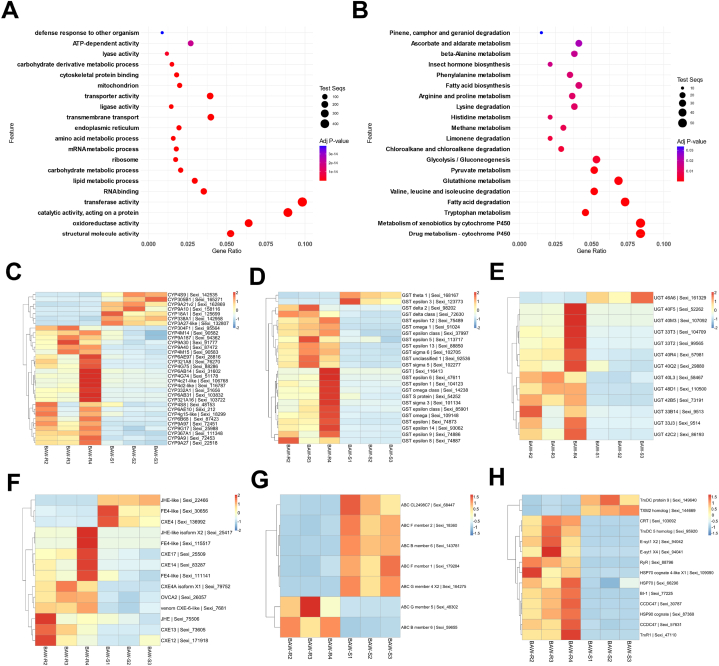
Functional enrichment analysis of differentially expressed genes (DEGs) and expression profiles of candidate genes. (A) Gene ontology (GO) enrichment analysis of DEGs using Fisher's exact test. Dots are arranged from the bottom in descending order of the adjusted p-value (Adj. P-value) with differences in color depending on the values. The size of the dots indicates the number of test sequences (Test Seqs). The Y-axis lists the feature as the enriched GO-slim terms, and the X-axis represents gene ratio calculated by dividing the number of DEGs by the size of the entire gene set. (B) Kyoto Encyclopedia of Genes and Genomes (KEGG) pathway enrichment analysis of DEGs using Fisher's exact test. The Y-axis and X-axis represent the third hierarchy of KEGG pathways and gene ratio, respectively. The criteria for the color and size of the dots are the same as in (A). (C–H) Expression patterns of candidate genes selected from DEGs across six samples including resistant and susceptible samples. The Y-axis contains annotated genes and unigene names (C): CYP; (D): GST; (E): UGT; (F): CCE; (G): ABC; (H): ER-Ca^2+^ homeostasis- and cell stability-associated genes.

### 3.6 Differential expression profile of potential chlorantraniliprole resistance-related candidate genes

We investigated the differential expression of detoxification enzyme groups — cytochrome P450 (CYP), glutathione S-transferases (GST), UDP-glucuronosyltransferases (UGT), carboxyl/cholinesterases (CCE), and ATP-binding cassette transporters (ABC) — known to play crucial roles in the development of diamide resistance and examined their expression patterns for each sample (Table S11; Fig. 5C–G). In total, 32 different P450 genes were successfully annotated, exhibiting differential expression within a range of −5.34 to 9.01 log2foldchange (LFC). Among these, 25 genes were upregulated, and 7 genes were downregulated (Fig. 5C). Additionally, 24 GST genes displayed differential expression within a range of −4.31 to 11.08 LFC, with 22 upregulated and 2 downregulated genes (Fig. 5D). Among the UGT gene family, 13 genes were differentially expressed within a range of −3.67 to 8.04 LFC, with 12 upregulated and 1 downregulated gene (Fig. 5E). The CCE gene family showed 14 DEGs within a range of −5.40 to 9.08 LFC, with 11 upregulated and 3 downregulated genes (Fig. 5F). Lastly, 7 ABC transporter genes were differentially expressed within a range of −4.23 to 5.82 LFC, with 2 upregulated and 5 downregulated genes (Fig. 5G). We investigated the RyR gene and found that its expression was significantly higher in the resistant strain, with a 7.43 LFC. Additionally, to identify the genes overcoming the calcium ion imbalance caused by diamide insecticides, we screened the differential expression profiles related to the ER and calcium ion homeostasis. Six genes were found to be upregulated (range of 2.2–12.2 LFC, Table S11). High expression levels of calreticulin (CRT), bax inhibitor 1 (BI-1), extended synaptotagmin-1 (E-syt1), and coiled-coil domain-containing protein 47 (CCDC47) were identified, with CRT showing the highest expression (LFC 12.21). Further analysis of CRT using STRING for protein–protein interactions revealed significant co-expression with protein disulfide isomerase, calreticulin/calnexin, thioredoxin, and the heat shock protein group (Fig. S1). Furthermore, functional enrichment analysis of these genes using STRING revealed that the proteins belonging to these groups are fundamentally involved in diverse cellular physiological processes such as endoplasmic reticulum unfolded protein response, response to endoplasmic reticulum stress, misfolded protein binding, and protein disulfide isomerase activity (Table S12). Based on these analyses, we discovered a total of 14 DEGs that may contribute to calcium ion homeostasis and cell stability against ER stress. The expression patterns of these genes are presented in Fig. 5H and Table S11. Consequently, the significantly upregulated genes were inferred to be involved in mitigating calcium ion imbalance within the ER, facilitating in protein folding, and ultimately protecting the cell.

### 3.7 Validation of candidate genes using qPCR

After identifying the upregulated genes in the resistant strain of *S. exigua* to diamide insecticide, we selected eleven highly expressed genes for qPCR analysis to further confirm their involvement in the enhancement of resistance in *S. exigua* to diamide. The selected genes for qPCR analysis include computational analysis identified upregulation of the genes within these interactions and those controlling calcium ion imbalance within the unigene. Eleven candidate genes— CYP9A40 (Unigene: Sexi_87472), glutathione S-transferase unclassified 1 (Sexi_92536), glutathione S-transferase epsilon 13 (Sexi_88850), calreticulin (Sexi_103092), bax inhibitor 1 (Sexi_77225), coiled-coil domain-containing protein 47 (Sexi_30787), extended synaptotagmin-1 (Sexi_94042), heat shock 70 kDa protein cognate 4-like (Sexi_109090), thioredoxin reductase 1, and mitochondrial (Sexi_47110), and RyR (Sexi_88796). The correlation between the expression pattern of diamide resistance-related candidate genes and the level of insecticide resistance can be considered by dividing them into 1) detoxification enzyme-related genes, 2) target site genes, 3) calcium regulation genes, and 4) HSP.

**Fig. 6:**
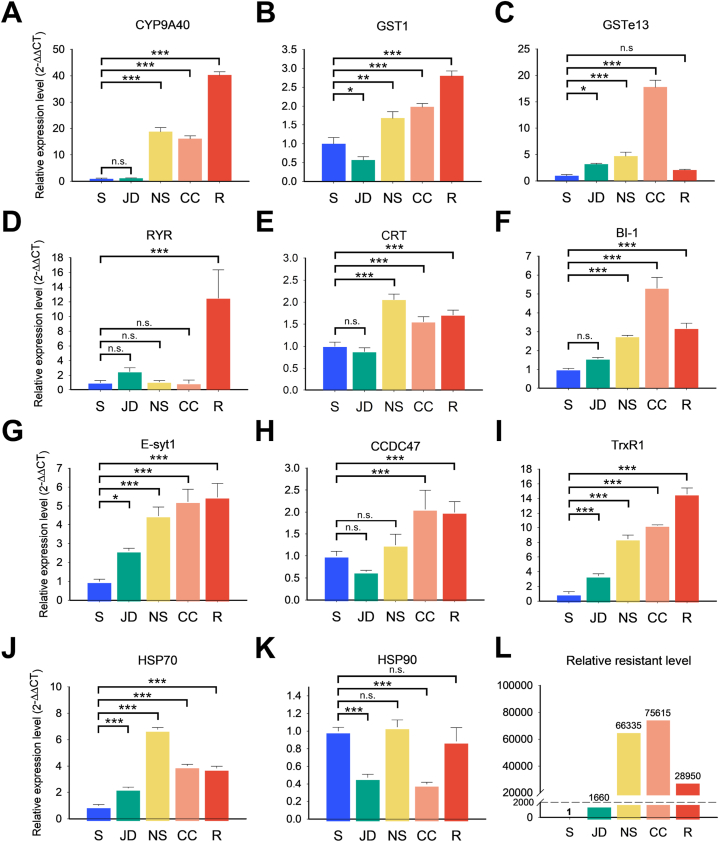
Relative expression level diversity of candidate genes examined by qPCR across five different populations with varying resistance levels. (A–K) Bar plots show the relative expression levels (2^−ΔΔCT^) of the candidate genes (A): *CYP9A40*; (B): *GST1*;(C): *GSTe13*;(D): *RYR*;(E): *CRT*;(F): *BI-1*;(G): *E-syt1*;(H): *CCDC47*;(I): *TrxR1*;(J): *HSP70* and (K): *HSP90* in the five different populations (X-axis). The qPCR results were normalized using *GAPDH* as the housekeeping gene. Relative expression levels were compared to those in the susceptible samples, calculated using the 2^−ΔΔCT^ method, and plotted on the Y-axis. The error bar represents the SD. Significant differences between populations were determined using one-way ANOVA followed by Tukey's HSD test then are indicated by asterisks (∗p < 0.05, ∗∗p < 0.01, ∗∗∗p < 0.001), and “n.s.” denotes non-significant differences. (L) The bar plot shows the relative resistance levels of the five different populations, with the susceptible population serving as the baseline (relative resistance ratio = 1). The resistance levels of the JD, NS, CC, and R populations are presented with the corresponding numerical values indicating the fold increase in resistance compared to the susceptible population.

First, the level of resistance is S < JD < R < NS < CC (Fig. 6L), and this result is the same as previously reported Han et al., [19,34]. 1) It was confirmed that genes related to detoxification enzymes, such as *CYP9A40* and two GSTs, were significantly positively correlated with the resistance and gene expression levels (Fig. 6A–C). 2) It was hard to find a correlation between the resistance and expression level of RyR, the target site. The RyR expression level was significantly high only in BAW-R, continuously exposed by chlorantraniliprole. There was little difference in the expression level of RyR from BAW-S and field populations such as NS and CC (Fig. 6D). 3)There are some differences in the expression level by population. Still, depending on the resistance level, genes such as CRT, BI-1, E-syt1, CCDC47, and TrxR1, which are involved in calcium regulation in ER, are differentially expressed (Fig. 6E–I). It was confirmed that the expression pattern was similar to the resistance level. 4) Between the two candidates' HSPs, the expression level of HSP70 increased depending on the resistance level (Fig. 6J). However, another HSP, HSP90, showed no correlation between the resistant ratio and expression level (Fig. 6K).

Therefore, it was confirmed that detoxification enzyme genes such as CYP and GST, as well as these genes involved in calcium regulation, are correlated with resistance development through the degree of resistance development and expression control.

## 4 Discussion

To protect themselves from the toxic effects of insecticides, insects can develop genetic changes through mutations or by increasing the expression of insecticide resistance-related genes. Consequently, researchers are investigating whether the primary mechanism enhancing insecticide resistance is due to mutations, changes in the expression of resistant-related genes, or a combination of both.

In this context, Han et al. (2023) did not observe a strong correlation between the I4790M mutation in the RyR and resistance in Korean diamide-resistant strain and field populations of *S. exigua* to diamide insecticide [19]. Conversely, Han et al. (2024) reported a strong correlation between resistance in the BAW-R strain and the expression of the *CYP9A40* gene, suggesting that elevated *CYP9A40* expression is a major factor contributing to resistance in this strain [34]. To further support this they conducted bioassay analysis using Piperonylbutoxide (PBO), an inhibitor of CYPs including *CYP9A40*, and found a decrease in the LC_50_ value for chlorantraniliprole in the BAW-R strain. These results indicate that the enhanced resistance is more likely due to changes in gene transcription rather than mutations. However, the bioassay results showed that a high level of resistance was still observed even after inhibiting CYP. From these findings, we can conclude that another resistance mechanism might be involved, beyond RyR and detoxification enzymes, potentially involving calcium regulation. Therefore, this study focused on the involvement of RyR, detoxification enzymes, and calcium regulation in enhancing resistance in both resistant strain and field population of *S. exigua* to diamide insecticide from a transcriptomic perspective. The purpose of studying these different resistance mechanisms was to identify the most crucial one, as the application of the *CYP9A40* inhibitor did not result in the expected significant decrease in the resistance.

The findings from GO and KEGG pathway enrichment analyses confirmed the overrepresentation of metabolism- and transfer-related DEGs using Fisher's exact test, suggesting that changes in the expression of various metabolic genes, including detoxification enzymes, potentially contribute to diamide resistance. To accurately interpret the distribution patterns of genes specific to resistance or susceptibility, orthologous analyses were performed, comparing 404 and 407 clusters for susceptible and resistant strains, respectively. Pathway enrichment analyses revealed that resistant strain possessed a larger gene pool involved in metabolism. We then screened ER and calcium ion regulatory gene expression profiles, focusing on genes countering the calcium ion imbalance induced by diamide insecticides and contributing to cellular stability. Since the resistance ratio (RR) was calculated based on that of BAW-S, gene expression was also compared based on the BAW-S. Consistent with previous studies [30,47,48], our findings suggest that generalist detoxification enzymes, including CYP and GST families, are upregulated in response to chlorantraniliprole. This upregulation likely enhances the detoxification and expulsion of the insecticide, supporting the observed resistance mechanisms [30,47,48]. The expression of RyR varies with developmental stages in some lepidopteran species, and diamides can induce its expression [49,50]. In this study, despite no significant differences in RyR expression between susceptible and field populations, the resistant (R) strain exhibited markedly higher expression levels (Fig. 6D). This suggests that continuous exposure to diamides under laboratory conditions may have led to RyR overexpression in the R strain, as previously suggested [50]. However, the contribution of RyR expression to the development of diamide resistance remains unclear and appears incomplete, as observed in other populations. RyR expression can vary among different species, and while it may play a role in resistance, it is not definitively correlated with resistance levels in field populations. Therefore, we extended our investigation to identify additional mechanisms that might contribute to the enhancement of resistance in both the resistant strain and field population in response to diamide insecticide. To this end, we conducted RNA-seq and qPCR analysis to examine the correlation between the expression of calcium-regulated genes and the resistance levels in both the resistant strain and field populations. Our findings revealed that genes involved in overcoming calcium ion imbalance and preventing cell death were also upregulated (Fig. 5, Fig. 6 and Table S11).

Our current findings align with previous studies they reported similar mechanisms. Upregulated calreticulin enhances the buffering capacity for Ca^2+^ within the ER, contributes to intracellular Ca^2+^ homeostasis, and aids in proper protein folding, thus alleviating ER stress [[51], [52], [53], [54]]. Upregulated E-syt1 helps stabilize membrane contact sites between the ER and plasma membrane upon ER Ca^2+^ depletion, facilitating rapid SOCE-mediated ER Ca^2+^ replenishment to overcome ER stress [55,56]. Upregulated CCDC47, also known as calumin, binds calcium ions with high capacity in its acidic ER luminal domain, preventing excessive Ca^2+^ efflux and playing a crucial role in intracellular Ca^2+^ exchange and cell fate signaling [57]. BI-1, an ER-located conserved cell death suppressor, inhibits the apoptosis signals induced by ER stress and sustained UPR, thus actively controlling ROS accumulation and PCD [[58], [59], [60], [61], [62]]. Upregulated TrxR1 catalyzes thioredoxin reduction, which is essential for redox regulation and mitigating oxidative stress during detoxification and ER stress [63,64]. Upregulated HSPs, particularly HSP70, likely play a crucial role in preventing the accumulation of misfolded proteins due to ER Ca^2+^ imbalance and assisting in proper protein folding, which may significantly contribute to overcoming ER stress induced by diamide insecticide [65,66].

**Fig. 7:**
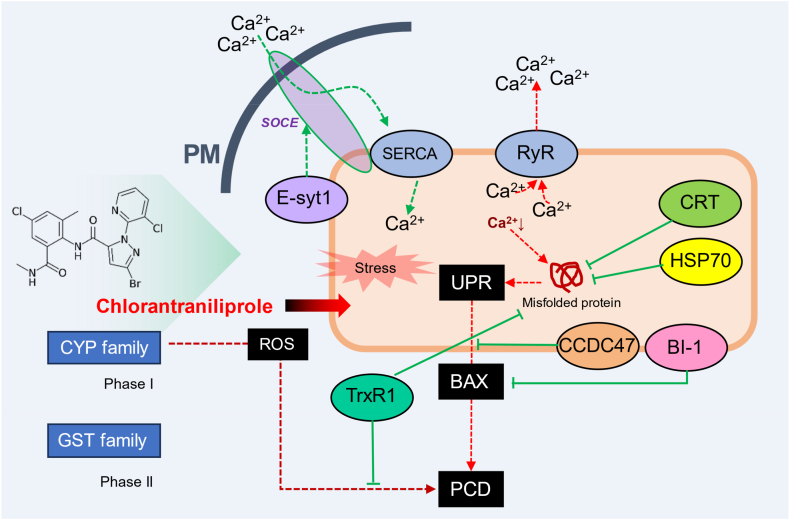
Proposed mechanisms of chlorantraniliprole resistance induction by potential genes regulating calcium ions. Chlorantraniliprole binds to the RyR, causing Ca^2+^ release and subsequent ER stress. Stress induces the unfolded protein response (UPR) and reactive oxygen species (ROS) production. CRT enhances Ca^2+^ buffering within the ER, contributing to protein folding and reducing ER stress. E-syt1 stabilizes membrane contact sites between the ER and PM upon ER Ca^2+^ depletion, facilitating SOCE-mediated Ca^2+^ replenishment. CCDC47 binds calcium ions in the ER, preventing excessive Ca^2+^ efflux and aiding in intracellular Ca^2+^ exchange and cell signaling. BI-1 inhibits apoptosis induced by ER stress and sustained UPR, actively controlling ROS accumulation and PCD. TrxR1 catalyzes thioredoxin reduction, essential for redox regulation and mitigating oxidative stress during detoxification and ER stress. HSP70 helps prevent the accumulation of misfolded proteins due to ER Ca^2+^ imbalance and assists in proper protein folding, contributing significantly to overcoming ER stress induced by chlorantraniliprole. Detoxification phase I and phase II including CYP and GST family metabolize chlorantraniliprole simultaneously. These processes collaborate to enhance cellular survival under stress conditions induced by chlorantraniliprole.

Overall, chlorantraniliprole enters cells and binds to the RyR target site, causing RyR to open and release calcium ions into the cytosol [7,8,11]. Disruption of metabolic processes such as protein folding, which require ER calcium ions, leads to ER stress and the activation of UPR mechanisms [67]. While UPR helps cells eliminate misfolded proteins and enhance protein folding capacity, sustained ER stress due to calcium depletion promotes ROS generation, activates pro-apoptotic proteins like BAX and induces apoptosis via the mitochondrial pathway [[68], [69], [70]]. The mechanisms of chlorantraniliprole resistance induced by the upregulation of potential genes are proposed in Fig. 7.

However, we emphasize that candidate genes that regulate calcium ions at the cellular level play a crucial role in developing chlorantraniliprole resistance based on bioinformatics analyses and relative expression experiments. Due to the limitations posed by an incomplete Lepidoptera database and constraints of bioinformatics analysis, the detailed functional interpretation of the genes covered in this study remains challenging. Furthermore, to determine the precise role of each candidate gene, *in vitro* and *vivo*-based functional validation approaches such as gene knockout will be required. Specific points need to be further verified, such as tissue-specific differences, and cross-validation among other lepidopteran species should be conducted to assess the consistency of observed phenotypes in further studies. However, it is a foundational basis for investigating candidate genes and their interactions that possibly confer chlorantraniliprole resistance.

Taken together, resistance to diamide insecticides of *S. exigua* suggests that it may have developed strategies to detoxify xenobiotics and counter calcium ion imbalances to enhance cellular survival, as inferred from *in silico* and *in vitro* analyses.

## 5 Conclusion

Bioinformatic analysis and qPCR confirmed the influence of calcium ion regulatory genes and previously identified metabolic factors associated with diamide insecticide resistance. Our findings propose that the complex mechanisms of calcium ion regulatory genes play an essential role in developing resistance to diamide insecticides such as chlorantraniliprole.

Building on previous findings that chlorantraniliprole resistance in *S. exigua* may be caused by more complex mechanisms than target site mutations and detoxification processes, this study utilizes RNA-seq transcriptome analysis to investigate novel insights into these underlying mechanisms. The results highlight the crucial role of genes involved in calcium ion homeostasis and ER stress regulation, such as calreticulin, E-syt1, and BI-1. Bioinformatic analysis and qPCR further confirmed their expression of these genes, and it is speculated that they may play a potential role in assisting the survival of *S. exigua* against chlorantraniliprole-induced calcium ion imbalance and cytotoxicity. These findings provide a more comprehensive approach to managing resistance to diamide insecticides by focusing on detoxification and calcium ion imbalance-induced cellular stress responses.

## CRediT authorship contribution statement

**Changhee Han:** Writing – review & editing, Writing – original draft, Methodology, Formal analysis, Data curation, Conceptualization. **Juil Kim:** Writing – review & editing, Supervision, Project administration, Conceptualization.

## Ethics statement

No endangered or protected species were used in the present study.

## Data availability statement

RNA-seq raw sequence data have been deposited in the National Center for Biotechnology Information (NCBI) Sequence Read Archive (SRA) under accession number SRX25348468–SRX25348476. Original codes are not included in this paper. Any additional requested information can be provided by the lead contact.

## Funding

This research was funded by the Cooperative Research Program supported by Agriculture Science and 10.13039/100006180Technology Development (project no. PJ01561902), 10.13039/501100003627Rural Development Administration, Republic of Korea, and by the Basic Science Research Program through the 10.13039/501100003725National Research Foundation of Korea (NRF) funded by the Ministry of Education, Republic of Korea (NRF-2021R1A6A1A03044242).

## Declaration of competing interest

The authors declare that they have no known competing financial interests or personal relationships that could have appeared to influence the work reported in this paper.
